# Using the common cold virus as a naturally occurring vaccine to prevent COVID-19: Lessons from Edward Jenner

**DOI:** 10.18632/aging.104166

**Published:** 2020-10-13

**Authors:** Federica Sotgia, Michael P. Lisanti

**Affiliations:** 1 Translational Medicine, School of Science, Engineering and Environment (SEE), University of Salford, Greater Manchester, United Kingdom

**Keywords:** coronavirus, viral spike glycoprotein, vaccine, common cold, COVID-19

## Abstract

Three recent papers published in Nature, Science and Cell, all present clear evidence that there is cross-reactive T-cell immunity between human coronaviruses (229E, NL63, OC43, and HKU1), linked with the common cold, and SARS-CoV-2, the causative agent of COVID-19. Can we use this information to design and build a new vaccine based on the less pathogenic, common cold coronaviruses, for the prevention of COVID-19? If we look at the history of medicine and vaccine development, from the point of view of Edward Jenner, the answer just might be yes.

Edward Jenner, was an English surgeon, who is credited with creating the first vaccine, in 1798, which was used to combat the Smallpox virus. Jenner employed the zoonotic Cowpox virus (as a live vaccine). Using the observation that milkmaids were somehow protected against Smallpox, he hypothesized that the pus from the milkmaid’s skin blisters could be used as a vaccine to inoculate other people, to protect against Smallpox. His successful clinical trial, of 23 patients, ultimately led the English Parliament to pass the Vaccination Act in 1840, making vaccination a new public health policy. His approach was used all over the world and ultimately led to the eradication of Smallpox by the WHO (World Health Organization) in 1980, nearly 40 years ago.

What can we learn today from Jenner’s observations that could be useful for designing a vaccine against SARS-CoV-2? Are there any less pathogenic viruses that could be used as a vaccine against SARS-CoV-2? The answer is probably yes.

For example, there are four human coronaviruses that are known to cause the common cold, namely 229E, NL63, OC43, and HKU1, which lead to mild upper respiratory infections (URI’s) [1–4]. According to the CDC, their route of transmission appears to be similar to SARS-CoV-2, but the onset of symptoms is quite mild in comparison. https://www.cdc.gov/coronavirus/general-information.html

All five viruses contain a viral spike glycoprotein (VSG), which is the main target of SARS-CoV-2 vaccine development world-wide.

One attractive hypothesis is that inoculation with the common cold coronavirus (229E, NL63, OC43, or HKU1) or, more likely, an attenuated version, could provide immunity against SARS-CoV-2. If that was the case, then we might already have a naturally-occurring vaccine at hand, that could soon be implemented, off the shelf.

To begin to test this hypothesis, we retrieved the protein sequences of the relevant viral spike glycoproteins from a variety of available databases, such as UniProt/FASTA, and analysed their shared protein sequence similarity and identity using BLASTP.

Table 1 summarizes the results of this brief analysis.

Based on this simple analysis, the viral spike glycoprotein of coronavirus OC43 appears to be the most similar to that of SARS-CoV-2, with nearly 38% identity and up to 53% similarity (Figure 1). In fact, the viral spike glycoproteins of coronavirus OC43 and HKU1 are also quite similar to each other, sharing 64% identity (Figure 2). So, both OC43 and HKU1 would possibly be good candidates for developing a potential vaccine to SARS-CoV-2.

**Figure 1:**
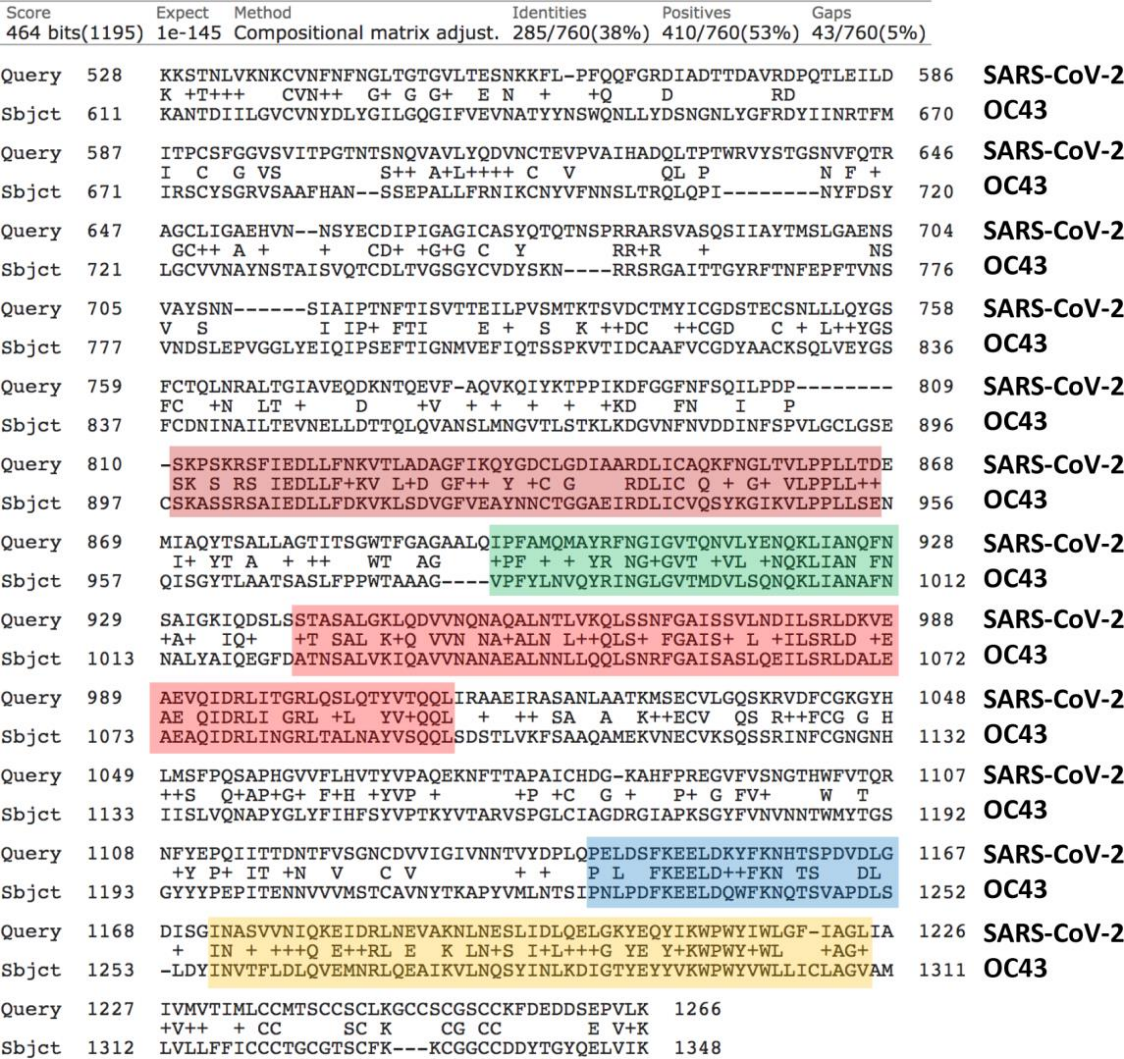
**Protein sequence alignments of the Viral Spike Glycoproteins (VSGs) from SARS-CoV-2 and the related Human Coronavirus OC43.** Areas of high sequence homology are highlighted in color, which may represent potentially shared epitopes for immune recognition. Generated using the online program BLASTP, by pairwise sequence analysis.

**Figure 2:**
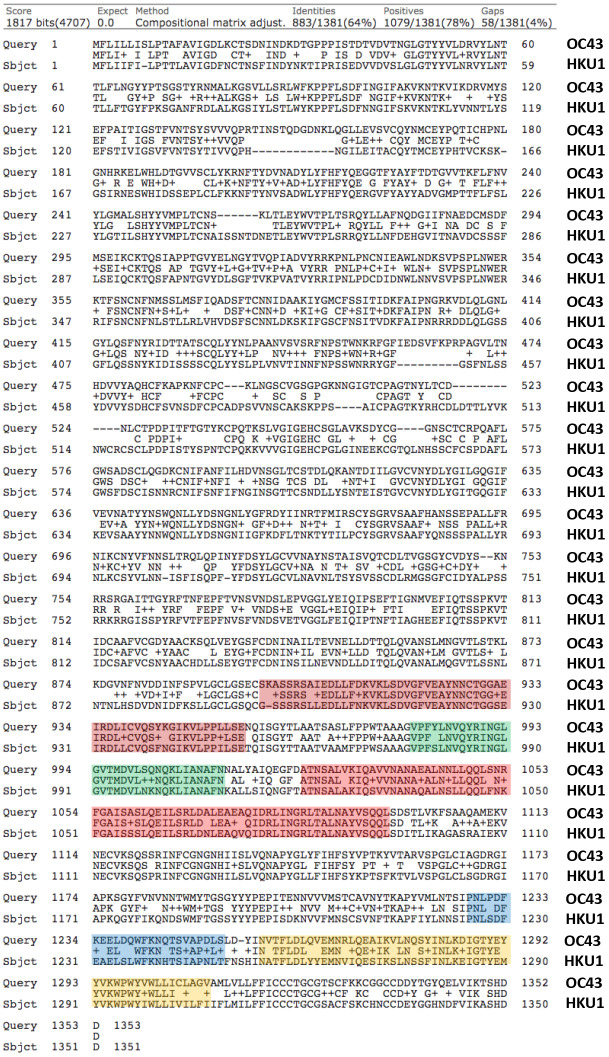
**Protein sequence alignments of the Viral Spike Glycoproteins (VSGs) from two related Human Coronaviruses, namely OC43 and HKU1.** Note the high homology between OC43 and HKU1, with up to 78% similarity. Generated using the online program BLASTP, by pairwise sequence analysis. The same potentially shared epitopes, highlighted in color in Figure 1, are also highlighted here, for comparison.

**Table 1:** Protein sequence identity of the viral spike glycoproteins of SARS-Cov-2 and the common cold corona viruses (229E, NL63, OC43, or HKU1).

**Common Cold VSG**	**SARS-Cov-2 VSG**
**229E**	27.78%
**NL6**	31.27%
**OC43**	37.65%
**HKU1**	36.66%

Is there any clinical evidence to support these assertions?

Three recent papers published in Nature, Science and Cell have begun to look at the existence of cross-reactive immunity in a variety of patient populations, especially patients infected with the SARS-CoV-2 (with frank COVID-19 or asymptomatic) and uninfected patients. The results are all quite encouraging, directly demonstrating cross-reactive T-cell immunity between SARS-CoV-2 and the existing known human cold coronaviruses (229E, NL63, OC43, and HKU1) [5–7]. One of the papers also detected cross-reactive serum IgG as well.

These reports clearly provide tantalizing clinical evidence for the feasibility of using a human cold coronavirus, such as attenuated OC43 or HKU1, as a potential vaccine for the prevention of COVID-19. What would Edward Jenner suggest, if he was living today?.

Further support for this idea has recently appeared in the popular press and was supported by data from the National Institutes of Health (NIH), because there is significant shared serological cross-reactivity between SARS-CoV-2, OC43 and HKU1 [8, 9].

Fortunately, two live coronaviruses, OC43 and 229E, associated with the common cold, are actually commercially available from the American Type Culture Collection (ATCC), which could greatly facilitate their potential use in new, off-the-self, vaccine development.

https://www.lgcstandards-atcc.org/products/all/VR-1558.aspx

https://www.lgcstandards-atcc.org/products/all/VR-740.aspx

Moreover, the VSGs from OC43 and HKU1, may also be sufficient to convey cross-reactive immunity, when recombinantly-inserted in another non-pathogenic viral vector, specifically designed for live or attenuated vaccine immunizations (Figure 3).

**Figure 3:**
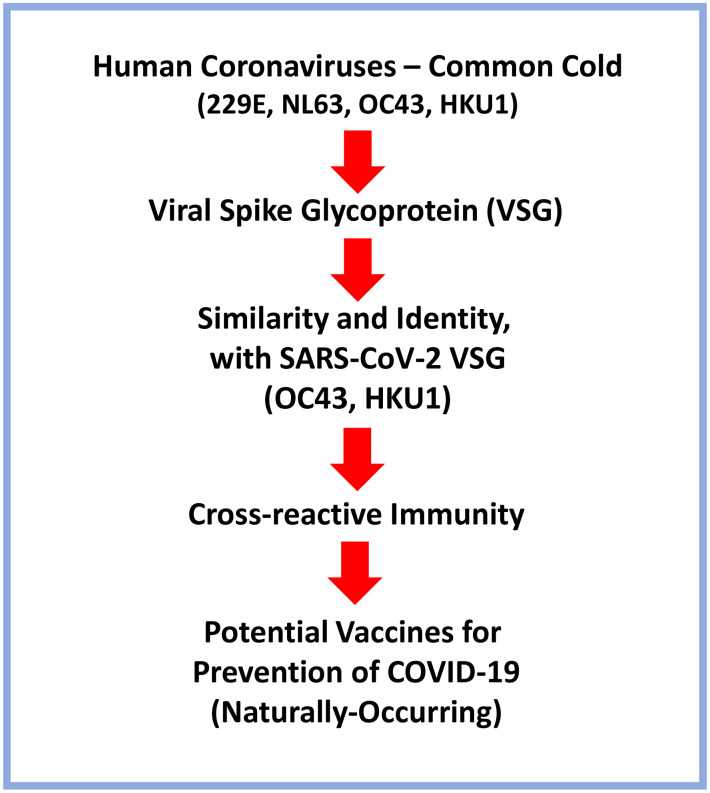
**Schematic diagram summarizing the possible use of Human Coronaviruses that cause the common cold as naturally-occurring vaccines for targeting SARS-CoV-2 and preventing COVID-19.** A brief flow-diagram is presented, outlining a vaccine development strategy.

Ultimately, this may be a safer approach, than using the VSG from SARS-CoV-2, which may have mild negative, or even pathogenic, side-effects. Only time will tell.

Nature may have already done the “experiment” or “clinical trial” for us, as so many people that are SARS-CoV-2 virus-positive, are asymptomatic and show evidence of cross-reactive immunity, to both SARS-CoV-2 and the common cold coronaviruses.

These findings have been independently confirmed now, in several different laboratories world-wide.

## UNIPROT accession numbers for 5 relevant protein sequences:

P0DTC2,

SPIKE_SARS2 Spike glycoprotein, Severe acute respiratory syndrome coronavirus 2

https://www.uniprot.org/uniprot/P0DTC2.fasta

Q6TUL7,

CVH22 Spike glycoprotein Human coronavirus 229E

https://www.uniprot.org/uniprot/Q6TUL7.fasta

Q6Q1S2,

SPIKE_CVHNL Spike glycoprotein Human coronavirus NL63

https://www.uniprot.org/uniprot/Q6Q1S2.fasta

P36334,

SPIKE_CVHOC Spike glycoprotein Human coronavirus OC43

https://www.uniprot.org/uniprot/P36334.fasta

Q0ZME7,

SPIKE_CVHN5 Spike glycoprotein Human coronavirus HKU1

https://ebi10.uniprot.org/uniprot/Q0ZME7.fasta

## Supplementary Material

Supplementary Note
