# Supplementary material for: Using the common cold virus as a naturally occurring vaccine to prevent COVID-19: Lessons from Edward Jenner
Source: Aging (Albany NY). 2020 Oct 13;12(19):18797–803. doi: 10.18632/aging.104166 (PMC7732292; doi:10.18632/aging.104166)
Supplement: Supplementary Note [file aging-12-104166-s001..pdf]

Note Added in Proof

After this ***Perspective Article*** was submitted for peer-review, another relevant paper appeared in the *British Medical Journal (BMJ)*, highlighting the role of human coronaviruses associated with the common cold in conferring cross-reactive immunity to SARS-CoV-2 in the world population.
https://www.bmj.com/content/370/bmj.m3563.full

Doshi P. Covid-19: Do many people have pre-existing immunity? The British Medical Journal. 2020; 370:m3563.
